# Effects of biophysical constraints, climate and phylogeny on forest shrub allometries along an altitudinal gradient in Northeast China

**DOI:** 10.1038/srep43769

**Published:** 2017-03-07

**Authors:** Han Sun, Xiangping Wang, Yanwen Fan, Chao Liu, Peng Wu, Qiaoyan Li, Weilun Yin

**Affiliations:** 1 College of Forestry, Beijing Forestry University, Beijing 100083, China

## Abstract

Whether there is a general allometry law across plant species with different sizes and under different environment has long been controversial and shrubs are particularly useful to examine these questions. Here we sampled 939 individuals from 50 forest shrub species along a large altitudinal gradient. We tested several allometry models with four relationships simultaneously (between stem diameter, height, leaf, stem and aboveground biomass), including geometric, elastic and stress similarity, and metabolic scaling theory’s predictions on small plants (MST_s_) and trees (MST_t_). We also tested if allometric exponents change markedly with climate and phylogeny. The predicted exponents of MST_t_, elastic similarity and stress similarity (models for trees) were not supported by our data, while MST_s_ and geometric similarity gained more support, suggesting the finite size effect is more important for shrub allometries than being a woody plant. The influence of climate and phylogeny on allometric exponents were not significant or very weak, again suggesting strong biophysical constraints on shrub allometries. Our results reveal clear differences of shrub allometries from previous findings on trees (e.g. much weaker climatic and phylogenic control). Comparisons of herbs, shrubs and trees along a same climatic gradient are needed for better understanding of plant allometries.

Whether there is a general allometry law across plant species with different sizes and under different environment has long been a focus in ecology^1^,^2^,^3^,^4^,^5^,^6^, and a number of hypotheses have been proposed to explore the underlying mechanisms. One group of hypotheses are based on assumptions of basic biophysical constraints on plant allometries and have provided testable predictions on scaling exponents (Table 1), including physical models (geometric^7^, elastic and stress similarity^8^,^9^) and biophysical ones (metabolic scaling theory, MST)^5^,^10^,^11^. The physical models consider some physical and geometric constraints of plants, such as isometrical scaling of organ length and radius (geometric similarity^7^), margin of safety of branches against mechanical failure (elastic similarity^8^,^9^) or the stress produced by wind pressure along the stem (stress similarity^8^,^9^). On the other hand, MST further considers biological constraints (e.g. maximization of metabolic rate) and predicts far more relationships than physical models. MST first proposed a model for tree allometries (MST_t,_ hereafter)^5^,^11^. However, subsequent MST studies demonstrated that the predictions for small plants (MST_s_) should be different. This is because small plants (e.g. herbs) lack some key features of trees: high stature, branching networks with a large number of branchings^12^,^13^,^14^, and large amounts of secondary xylem^15^. For instance, for small plants MST predicted that (Table 1): *H* (height) ∝ *D* (Diameter), *M*_L_ (leaf mass) ∝ *M*_S_ (stem mass), *M*_A_ (aboveground mass) ∝ *D*^3^ and *M*_A_ ∝ *H*^3^ (for details, see Appendix 1).

These models are important in exploring the general allometric laws for plants^5^,^11^,^16^,^17^,^18^ and have attracted many research interests (especially MST). However, these models have also been controversial^6^,^18^,^19^,^20^,^21^,^22^. Thus further tests are still needed across species groups and regions, and some limitations in previous tests require attention. First, each model makes predictions for multiple allometric relationships (Table 1), which theoretically need to be tested simultaneously. However, many studies have tested only one or two predicted exponents of the models, which may lead to bias in rejecting the models^18^. Second, most tests have been conducted using data from trees^5^,^9^,^20^,^23^. Although some studies have tested MST with data including plants from herbs to large trees^15^,^22^,^24^,^25^, allometry data for shrubs are still rather limited compared with trees^26^.

Shrubs are particularly useful to test these models and to examine the size-dependence of plant allometries. On one hand, shrubs differ from herbs in that they have well-developed secondary xylem; thus they may support MST_t_. On the other hand, shrubs differ from trees in that they typically have lower numbers of branches because of low stature (the finite size effects^12^), so they may support MST_s_. Some studies have suggested that the elastic and stress similarity models were suitable for trees while geometric similarity suitable for small plants (though there is still no consensus)^8^,^27^,^28^. Since the stature of shrubs is more similar to herbs than trees and MST suggests that the finite size effects are important for plant allometries^12^,^29^, we predicted that our shrub data will provide more supports to MST_s_ and the geometric similarity model, instead of MST_t_, elastic similarity or stress similarity.

In contrast to biophysical models that predict a single exponent for each relationship (Table 1), another group of studies have related allometries to environmental gradients and suggested that allometric exponents change markedly with climate and site quality^21^,^23^,^30^,^31^. For instance, the allometric exponent between mean tree height and mean biomass per stem (commonly used to test MST_t_^16^,^17^) was found to be significantly affected by climate and dominant species in northeast China^32^. The scaling exponent of individual tree diameter with height has also been found to vary remarkably along climate gradients^2^,^23^,^30^,^31^. Meanwhile, the extent to which phylogeny affects allometric exponents is also controversial. The supporters of MST showed that allometric exponents do not differ much across a wide range of phylogenetic affiliations from herbs to trees^4^,^16^,^33^,^34^,^35^. However, others have found that allometric exponents differ among phylogenetic groups, species, and even intra-specifically^18^,^21^,^23^,^31^. Thus, the influence of climate and phylogeny on plant allometries still needs further examination.

Few studies have examined allometries of forest shrubs in relation to large climatic gradients. In this study, we sampled shrubs along the altitudinal gradient of Mt. Changbai in northeast China to examine three questions as follows: (1) Does shrub allometry conform to MST_s_ and geometric similarity rather than MST_t_, elastic similarity or stress similarity? We tested these models with four allometric relationships simultaneously: height vs. stem diameter, leaf vs. stem biomass, aboveground biomass vs. height and vs. diameter. (2) Are shrub allometric exponents strongly affected by climate gradients and phylogeny (as observed by previous studies on trees), or only slightly affected as predicted by MST? If the latter is true, this may suggest biophysical constraints plays an important role in shaping the allometries of forest shrubs.

## Results

### Testing the biophysical models

**Figure 1:**
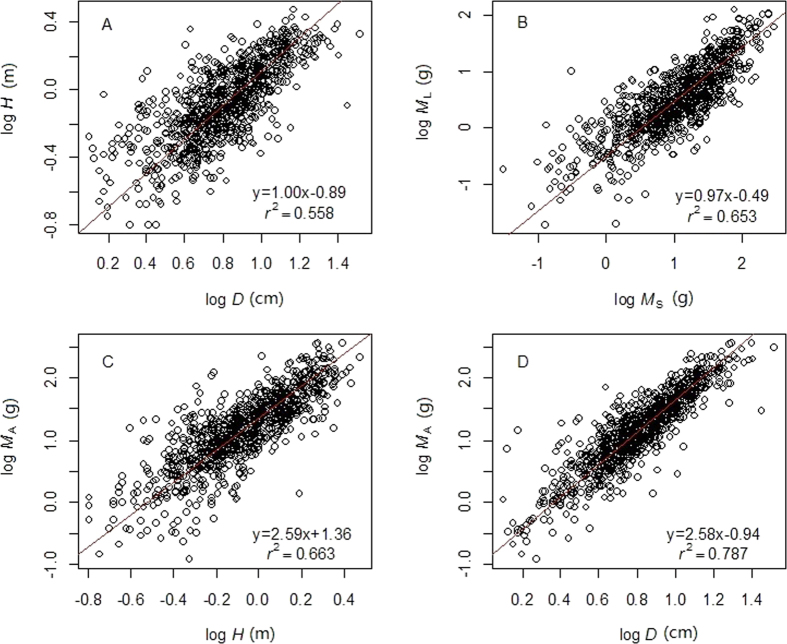
Allometric relationships for data from all plots combined, using standard major axis regression. (**A**) Height (*H*, m) vs. diameter (*D*, cm). (**B**) Leaf biomass (*M*_L_, g) vs. stem biomass (*M*_S_, g). (**C**) Above-ground biomass (*M*_A_, g) vs. *H*. (**D**) *M*_A_ vs. *D*.

To test the models, we used data from all plots together (Fig. 1) and standard major axis (SMA) regression to examine whether the model predicted exponents were within the 95% confidence intervals (CIs) of SMA slope, for both raw data and phylogenetic independent contrasts (PIC) data (see Methods).

The five biophysical models differed markedly in their consistency with the observed data (Table 1). As predicted, the MST_t_, elastic similarity and stress similarity models were not supported. For stress similarity, only one prediction (*M*_A_-*D*) was consistent with our raw data.

All four predictions of MST_s_, and all the three predictions of geometric similarity, were supported in the PIC data. Some predictions of the two models were not consistent with the test using original data. Since the PIC test is more robust compared with the test with original data (see Methods), we concluded that our data provided more support to MST_s_ and geometric similarity than the other three models.

### Influence of climate and phylogeny on allometric exponents

Three of the SMA slopes of the original and PIC data were not significantly different (Table 1). This suggests that phylogeny has no influence on most allometric exponents. Further, when the allometric exponents and intercepts of the 25 plots were related to mean annual temperature (MAT) and precipitation (MAP) along the altitudinal gradient (Table 2), only two of the 16 correlations were significant, suggesting that shrub allometries are also only weakly affected by climate.

These results were confirmed by a more detailed analysis with mixed-model analysis of variance (ANOVA) (Table 3). For allometric exponents (i.e. the interaction terms), climate had little influence. Only MAP had a significant relationship with the *M*_A_-*D* allometry, and MAT had a significant relationship with *M*_A_-*H*, but both explained negligible proportions of variance (0.12% and 0.39%, respectively). The effect of site (i.e. potential effect of other local conditions) was significant for all four allometric exponents, but still explained only 0.52% to 1.70% of variance. For the *H*-*D* and *M*_L_-*M*_S_ relationships, none of the phylogenetic terms were significant (at *P* < 0.05). For *M*_A_-*H* and *M*_A_-*D*, only one term (e.g. the “log *H*: species” term) was significant, but again explained only 0.53% to 0.61% of variance. When the phylogenetic terms were replaced with taxonomic groups, the results were similar (Appendix 2). In summary, the effects of both climate and phylogeny on allometric exponents were very weak (if there were any) for the forest shrubs in this study.

For allometric constants (i.e. the effect of environmental and phylogenetic variables themselves), more variables were significant and explained more variation compared with the interaction terms (Table 3). This suggest that climate and phylogeny have some influence on shrub allometries, but mainly through allometric constants rather than exponents, which is consistent with a prediction of MST^18^,^33^,^35^. However, since most of the correlations of intercepts with climate were not significant (Table 2), this issue requires examination in future studies. Consequently, we will not comment on it further.

## Discussion

Since the biophysical allometry models (Table 1) were proposed, much effort have been devoted to test them^18^,^27^,^36^. Each model has gained some level of support but has also been controversial.

Here we tested the models using a large shrub dataset along a large altitudinal gradient. Similar as MST_t_, the elastic and stress similarity models were originally developed for tree allometries^8^, and were then extended to other plants and even animals. As we expected, they were not supported by our shrub forest data (Table 1). Though stress similarity have one prediction (*M*_A_-*D*) supported by the raw data, but other predictions could not be supported simultaneously. This implies that studies that use only one or two allometric relationships to test these biophysical models could well have reached stronger conclusions than warranted.

Metabolic scaling theory now days can account for different models of scaling (geometric, elastic similarity etc.) and suggests that allometric exponents change continuously with plant size rather than being constant as misunderstood in the literature^12^,^37^,^38^. MST suggests that plant vascular networks are self-similar and has derived that: *l*_*k*_ ∝ *r*_*k*_^α^ (where *l*_*k*_ and *r*_*k*_ is the length and radius of branches at the *k*^th^ level in the branching network). When α = 2/3 (i.e. elastic similarity) for all *k* (i.e. self-similar), then the predictions for trees (MST_t_) can be derived. For small plants, however, α = 1 (i.e. geometric similarity) for all *k*, which leads to the predictions of MST_s_ (for details see ref. 29). Price *et al*. (2007) further proposed a new version of MST^38^. By relaxing some assumptions of MST_t_ and explicitly addressing variation in network design, they showed that allometric exponents should change continuously with plant size, and the predictions of geometric similarity, elastic and stress similarity and the fractal branching model (i.e. MST_t_) all fall along this continuum from the smallest herbs to the tallest trees. Shrubs are expected to be located near the end for small plants along this continuum because of finite size effects (see Introduction). This prediction was confirmed by our data, which provided more support to MST_s_ and geometric similarity compared with the other three models. Meanwhile, other MST_s_ predictions have also received supported from previous studies on small plants^15^,^26^. It seems that MST provides a basis to develop a general theory of plant allometry in the future.

While numerous allometric studies have been conducted, most were local-scale. Relative few papers have examined whether and how the scaling relationships of trees vary along climatic gradients^23^, and such studies are even rarer for shrubs. Despite this, existing evidence for trees indicates that allometric exponents change significantly with climate. This is true for other allometries as well as *H*-*D* scaling, and has been found in studies from regional to continental scales^2^,^23^,^30^,^31^,^32^,^39^,^40^.

Even so, in this analysis on shrubs we found that the exponents of four scaling relationships were largely unaffected by climate (Tables 2 and 3). We covered a large climate gradient from timberline to low-elevation temperate forests, which was comparable to that of Wang *et al*.^30^ in terms of temperature; but they found strong climatic control of tree *H*-*D* allometry in northeast China. Thus, our results suggest that there are real differences between shrubs and trees in the modulators of allometries.

For the tree growth form, the tall stature lead to great difficulty in water transportation from root to canopy (i.e. hydraulic resistance)^41^,^42^. While tapering of conduits from stem base to top is found to be an effective compensation mechanism^43^, it may only partially compensate for the increase in hydraulic resistance with tree height^44^. Many studies have found that the hydraulic efficiency of tree xylem is reduced in drier or colder climates^45^,^46^. Thus, trees have to adjust the relative biomass allocated for stem diameter, height and branch growth in response to climate gradient^23^,^30^. However, the situation is much different for shrubs. First, the low stature of shrub means that it has much lower hydraulic resistance than trees. Second, studies on tree and shrub physiology showed that conduit tapering can compensate for most hydraulic resistance for low stature plants but not for tall trees^44^,^47^,^48^. Thus, for forest shrubs under humid climate (e.g. in our study) there seems not much difficulty in vertical water transportation. This may be an important reason why the allometric exponents in our study were found to be insensitive to climate. However, our findings may not be easily extended to arid shrub or desert biomes, where very low soil water potential may still cause vertical water transportation to be difficult for shrubs.

Our results indicate that the effect of phylogeny on shrub allometries seem to be also very weak (Tables 1 and 3). This is again in contrast to studies on trees that revealed clear allometric differences among phylogenetic groups^18^,^21^,^23^,^31^,^49^. Why shrubs and trees may differ is still not clear and deserves further study. However, it seems that shrubs provide more support than trees to a prediction of metabolic scaling theory that allometric exponents were not sensitive to either environment or phylogeny^3^,^16^,^33^. Similarly, a recent study on understory shrubs in subtropical forests of China found that the scaling exponent of above- vs. below-ground biomass was consistent with MST for small plants, and was also insensitive to environmental variation and difference in species composition^26^. These results suggest that there are strong biophysical constraints on shrub allometries. Climate and phylogeny may play a secondary role in addition to biophysical constraints, and thus their effects on allometric exponents may be only obvious for trees.

Our result that the allometric exponents of forest shrubs are largely un-affected by climate gradient and phylogeny is important. Empirically, it helps to define numerically the boundary conditions for biomass allocation patterns across diverse shrub species and climate conditions^50^, and thus may contribute to a better estimation of forest understory carbon pools. The finding is also theoretically important because, it suggests that basic biophysical constraints are crucial in shaping shrub allometries, and that the modulators for small plant allometry differ markedly from that of trees. These differences between shrubs and trees may arise partly because of the difficulty in vertical water transportation for trees; and may also because hasher climate limits tree height^30^,^42^ which in turn leads to size-related changes in allometries^37^,^38^. Further, for trees the change of allometric exponents across climate gradients can be partially caused by environmentally driven recruitment limitation and successional status^39^. Which of these mechanisms is more important for plant allometries remains unclear. Thus, systematic comparisons of herb, shrub and tree allometries across the same climatic gradient are needed to address these questions.

## Methods

### The study area

Mt. Changbai (41°43′–42°26′N, 127°42′–128°17′E) is located at the border between Northeast China and North Korea. It is the highest mountain (2691 m) in Northeast Asia and all latitudinal forest zones in northeast China can be found along its altitudinal gradient. Thus, Mt. Changbai provides an ideal location to test ecological hypotheses related to climatic gradients^51^,^52^.

The regional climate is controlled by the East Asian monsoon, with warm summers, cold winters, abundant precipitation and a short growing season. With increasing altitude from the base to the top of Mt. Changbai, mean annual temperature decreases from 4.9 to −7.3 °C, and mean annual precipitation increases from 600 to 1340 mm^53^. As a result of the climatic gradients, three forest zones are distributed along the altitudinal gradient: (1) <1100 m: mixed broad- & needle-leaved forest, composed of *Pinus koraiensis* and broad-leaf species; (2) 1100–1700 m: evergreen needle-leaved forest, composed of *Picea jezoensis* and *Abies nephrolepis*; (3) 1700–2000 m: *Betula ermanii* forest^54^.

### Sampling method and climate data

We set 25 plots of 20 m × 30 m along the altitudinal gradient, ranging from 450 m to 1900 m a.s.l., covering all the vertical forest zones of Mt. Changbai. We selected ca. 10 individuals of variable sizes of each dominant shrub species in the understory of each plot. We made efforts to avoid multi-stem individuals, and sampled only individuals with distinct main stem at the base (i.e. individuals that the base diameter can be measured). In a few cases, when this is not possible, we measured each stem separately for a multi-stem individuals, and other variables were also measured separately. We measured basal stem diameter and vertical height before harvesting the individuals. The dried weights of leaf and stem for each individual were determined by oven-drying for 72 h at 60 °C. A total of 939 individuals belonging to 50 shrub species (from 31 genera and 18 families) were sampled. Statistic descriptions of these variables are listed in Table 4.

The climatic data of the plots were estimated using the models developed by Wang *et al*.^55^. The models were fitted with data from climate stations across the Changbai Mountains and adjacent regions, in which monthly mean temperature and precipitation were estimated with linear models using latitude, longitude and altitude as predictors. Wang *et al*.^55^ validated their models using independent climate data, and have shown that the estimated climates were sufficiently accurate^55^. We initially calculated six climate variables for each plot, including mean annual temperature and precipitation (MAT and MAP, respectively), mean temperature of the coldest month, warmth index and coldness index, and annual range of temperature. A preliminary PCA analysis indicated that MAT and MAP represent the two major axes of climatic variation, and other climate variables were highly correlated with them. To avoid collinearities among predictors, we used only MAT and MAP in statistical analyses.

### Phylogenetic tree and phylogenetic analyses

We constructed a phylogenetic tree for the 50 shrub species in this study to examine the effect of phylogeny on shrub allometries. The tree topology was built with the online program Phylomatic 2^56^, using the “Maximally resolved seed plant tree (version R20091110)” based on the supertree of the Angiosperm Phylogeny Group III (APG III 2009). In many cases, the Phylomatic program treated genera as polytomies within their families while species as polytomies within their genera. We searched for phylogenies in literatures^57^,^58^,^59^ and online databases (www.timetree.org), and the polytomies were substituted with published phylogenies whenever available. The branch lengths of the phylogenetic tree were determined using the BLADJ program^56^, with the nodes ages available in Wikström *et al*.^60^ and other sources^57^,^58^,^59^ including www. timetree.org.

**Figure 2:**
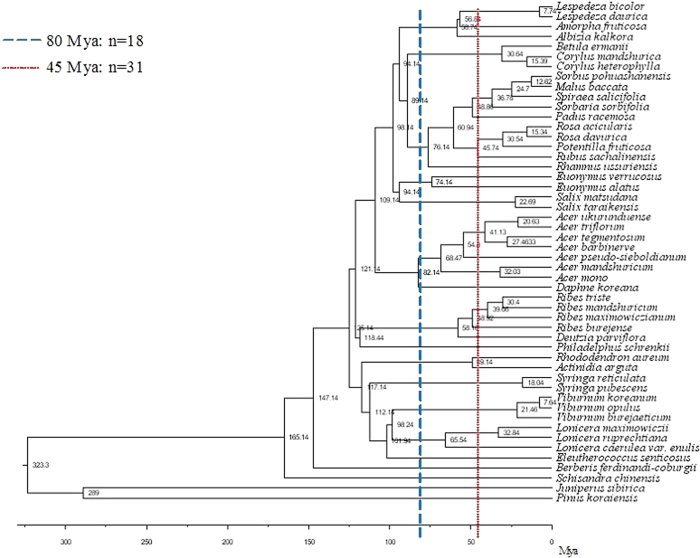
Phylogenetic tree for the 50 species in this study, showing the phylogenetic divisions at the 80 and 45 Mya. *n*, number of divisions.

We used the phylogenetic tree (Fig. 2) for two analyses: (1) phylogenetic independent contrasts (PIC)^61^,^62^. Closely related species tend to have similar traits, thus there may be non-independence of data due to phylogeny when analyzing the relationship between traits across species. To test the predicted allometric exponents of the biophysical allometry models more robustly, we created PIC for each allometric variable (*H, D, M*_L_, *M*_S_ and *M*_A_) using the Phylocom program^56^. We re-analyzed the allometric relationships using PIC (i.e. data when phylogenetic signals have been removed), in addition to the allometric analyses using original data.

(2) While the PIC approach is effective in removing phylogenetic signal, the method is not effective for examining how allometric relationships were affected by phylogeny and environmental factors together. For this purpose we created phylogenetic groups using the method described in He *et al*.^63^, by “cutting” the phylogenetic tree at 80 and 45 Mya (Fig. 1). We chose 80 and 45 Mya because they resulted in the same number of divisions (18 and 31, respectively) as the family or generic numbers of our species^63^. The phylogenetic groups thus created were then used as categorical variables in multivariate models of allometric relationships. This method has a great advantage that the relative effects of phylogenetic and environmental factors could be quantified and compared^63^,^64^. In this analysis, the non-independence of data (caused by phylogeny and site) was dealt with using mixed-model ANOVA, which will be described below.

### Statistic analyses

We examined four allometric relationships simultaneously for a better test of the biophysical allometry models: height (*H*) vs. diameter (*D*), leaf (*M*_L_) vs. stem biomass (*M*_S_), aboveground biomass (*M*_A_) vs. height, and *M*_A_ vs. *D*. All five variables were log-transformed prior to analysis. Allometric exponents and constants and their 95% confidence intervals (CIs) were obtained through linear regression of log-X and log-Y, a standard method in allometric analysis^17^. We used standard major axis (SMA) regression instead of ordinary least squares regression, because the latter can underestimate regression slope when both X and Y are measured with error. SMA analyses were conducted with the R package SMATR.

To test the biophysical models we used data from all plots to fit the four allometric relationships. For each relationship we examined whether the 95% CIs of the SMA slope included the predicted values of the biophysical models, using both the original and PIC data. To test the effect of phylogeny on allometries, we examined whether the SMA slopes of PIC were significantly different from that of original data, using the likelihood ratio test. If true, this indicates that phylogenetic relatedness significantly influences an allometric exponent.

We also fitted allometric relationships for each plot and correlated the plot-level SMA slopes and elevations with the climate indices of the plots to examine whether allometric relationships changed with climate. However, for a more detailed examination of the potential influence of environment and phylogeny on shrub allometries, we used general linear models (GLMs) and mixed-model ANOVA. For each allometric relationship, we explained log-Y using log-X together with other explanatory terms as follows: (1) climate (MAT and MAP); (2) site (i.e. the plots) was used as a categorical variable to account for other sources of environmental variation (e.g. canopy openness, soil fertility, etc.)^65^,^66^; (3) phylogenetic or taxonomic groups. Phylogenetic groups included three nested terms mentioned above: the 80 Mya and 45 Mya phylogenetic groups, and species. Taxonomic groups included family, genus and species. (4) The interactions between log-X and climate, site, and phylogenetic terms were included in GLMs to examine whether the regression slopes (i.e. allometric exponents) were significantly affected by environment and phylogenetic/taxonomic relationships.

In these analyses, climatic variables were nested within sites, and thus lead to non-independence of data. This problem was resolved by treating MAT and MAP as fixed factors while site as random effect in mixed-model ANOVA. The phylogenetic or taxonomic terms were also nested (e.g. genera nested within families). Thus, for the 80 Mya phylogenetic group (or family) the 45 Mya group (or genus) was used as the random effect; whereas for the 45 Mya group (or genus) the species term was treated as the random effect. Similarly, the interaction terms of climate and phylogeny with log-X were treated in a similar way (for details, see ref. 65). All statistical analyses were performed with R 3.3.1^67^.

## Additional Information

**How to cite this article**: Sun, H. *et al*. Effects of biophysical constraints, climate and phylogeny on forest shrub allometries along an altitudinal gradient in Northeast China. *Sci. Rep.* **7**, 43769; doi: 10.1038/srep43769 (2017).

**Publisher's note:** Springer Nature remains neutral with regard to jurisdictional claims in published maps and institutional affiliations.

## Supplementary Material

Supplementary Appendix 1

Supplementary Appendix 2

## Figures and Tables

**Table 1:** Testing the predicted allometric exponents of five biophysical models, using log-transformed data and standard major axis (SMA) regression.

	*H*-*D*	*M*_L_-*M*_S_	*M*_A_-*H*	*M*_A_-*D*
**Theory predictions**				
MST_t_	2/3	3/4	4	8/3
MST_s_	**1**	**1**	**3**	**3**
Geometric similarity	**1**	**—**	**3**	**3**
Elastic similarity	2/3	**—**	4	8/3
Stress similarity	1/2	**—**	5	5/2
**SMA slope** (**95% CIs**)				
Original data	1.00 (0.95–1.04) r^2^ = 0.558	0.97 (0.93–1.00) r^2^ = 0.653	2.59 (2.50–2.69) r^2^ = 0.663	2.58 (2.50–2.66) r^2^ = 0.787
PIC data	1.05 (0.82–1.34) r^2^ = 0.286	1.05 (0.87–1.28) r^2^ = 0.558	2.97 (2.36–3.72) r^2^ = 0.393	3.11 (2.73–3.53) r^2^ = 0.809
P value for likelihood ratio test	0.686	0.376	0.245	0.006**

For each allometric relationship, we report the SMA slope and 95% confidence intervals (CI) for both original data and phylogenetic independent contrasts (PIC) data. The theory prediction is underlined if it is within the 95% CI of the SMA slope for the original data, while in bold faced if supported by the PIC data. Abbreviations: *H***-***D*, height vs. stem diameter; *M*_L_-*M*_S_, leaf vs. stem mass; *M*_A_-*H*, aboveground mass vs. height; *M*_A_-*D*, aboveground mass vs. diameter. If the *P* value for the likelihood ratio test <0.05, then the SMA slope for PIC data is significantly different from that of the original data **P < 0.01.

**Table 2:** Correlations between climate and the SMA slopes and intercepts for each allometric relationship of the 25 plots.

	*H*-*D*		*M*_L_-*M*_S_		*M*_A_-*H*		*M*_A_-*D*	
	Slope	Intercept	Slope	Intercept	Slope	Intercept	Slope	Intercept
MAT	−0.14	0.22	−0.23	0.60**	0.51**	−0.04	0.31	−0.31
MAP	−0.01	−0.32	−0.24	0.13	−0.13	0.34	−0.08	−0.07

MAT and MAP, mean annual temperature and precipitation, respectively. **P < 0.01.

**Table 3:** Summary of mixed-model ANOVA for the effects of climate, site and phylogenetic groups on shrub allometric relationships.

	df	MS	P	%SS		df	MS	P	%SS
*H*-*D*					*M*_L_-*M*_S_				
log *D*	1	27.96	0.000	55.81	log *M*_S_	1	231.67	0.000	65.27
MAT	1	1.81	0.002	3.61	MAT	1	11.98	0.011	3.37
MAP	1	0.39	0.111	0.78	MAP	1	8.37	0.031	2.36
Site	22	0.14	0.000	6.22	Site	22	1.57	0.000	9.72
80 Mya	17	0.16	0.046	5.28	80 Mya	17	0.87	0.022	4.14
45 Mya	12	0.06	0.900	1.41	45 Mya	12	0.27	0.419	0.91
Species	17	0.12	0.000	4.16	Species	17	0.24	0.000	1.17
log *D*:MAT	1	0.07	0.187	0.13	log *M*_S_:MAT	1	0.00	0.940	0.00
log *D*:MAP	1	0.02	0.480	0.04	log *M*_S_:MAP	1	0.01	0.841	0.00
log *D*:Site	22	0.04	0.000	1.56	log *M*_S_:Site	22	0.17	0.000	1.05
log *D*:80Mya	17	0.03	0.353	0.91	log *M*_S_:80 Mya	17	0.09	0.060	0.43
log *D*:45Mya	11	0.02	0.395	0.47	log *M*_S_:45Mya	11	0.04	0.398	0.11
log *D*:Species	17	0.02	0.065	0.64	log *M*_S_:Species	17	0.03	0.878	0.15
Residuals	798	0.01		19.00	Residuals	798	0.05		11.32
***M***_**A**_**-*****H***					***M***_**A**_**-*****D***				
log*H*	1	222.93	0.000	66.33	log*D*	1	264.42	0.000	78.67
MAT	1	2.49	0.210	0.74	MAT	1	0.25	0.521	0.07
MAP	1	6.21	0.054	1.85	MAP	1	0.11	0.675	0.03
Site	22	1.50	0.000	9.79	Site	22	0.58	0.000	3.79
80 Mya	17	0.59	0.080	3.00	80 Mya	17	0.56	0.015	2.83
45 Mya	12	0.27	0.119	0.94	45 Mya	12	0.16	0.555	0.56
Species	17	0.14	0.000	0.72	Species	17	0.17	0.000	0.86
log *H*:MAT	1	1.30	0.036	0.39	log *D*:MAT	1	0.18	0.149	0.05
log *H*:MAP	1	0.39	0.235	0.12	log *D*:MAP	1	0.41	0.033	0.12
log *H*:Site	22	0.26	0.000	1.70	log *D*:Site	22	0.08	0.028	0.52
log *H*:80 Mya	17	0.12	0.474	0.62	log *D*:80 Mya	17	0.08	0.940	0.41
log *H*:45 Mya	11	0.12	0.407	0.38	log *D*:45 Mya	11	0.19	0.000	0.61
log *H*:Species	17	0.10	0.013	0.53	log *D*:Species	17	0.02	0.982	0.10
Residuals	798	0.05		12.90	Residuals	798	0.05		11.35

Climate includes mean annual temperature (MAT) and precipitation (MAP). Phylogenetic groups include three nested terms: 80 Mya and 45 Mya phylogenetic divisions, and species (Fig. 1). Allometric variables were log-transformed prior to analysis.

**Table 4:** Statistic descriptions of variables.

	Units	n	Mean	Median	s.d.	Max	Min
*D*	cm	939	7.41	6.51	3.99	33.02	1.25
*H*	m	939	0.95	0.83	0.49	2.98	0.16
*M*_S_	g	939	23.81	11.61	33.70	295.21	0.03
*M*_L_	g	939	7.78	2.96	13.82	125.24	0.02
*M*_A_	g	939	31.59	15.45	44.98	357.73	0.12

n, number of samples. s.d., standard deviation; *D*, diameter. *H*, height. *M*_S_, *M*_L_ and *M*_A_, biomass of stems, leaves and above-ground.
