# Supplementary material for: Effects of biophysical constraints, climate and phylogeny on forest shrub allometries along an altitudinal gradient in Northeast China
Source: Sci Rep. 2017 Mar 7;7:43769. doi: 10.1038/srep43769 (PMC5339776; doi:10.1038/srep43769)
Supplement: Supplementary Appendix 1 [file srep43769-s1.doc]

**Effects of biophysical constraints, climate and phylogeny on forest shrub allometries along an altitudinal gradient in** **Northeast China**

Authors: Han Sun, Xiangping Wang^*^, Yanwen Fan, Chao Liu, Peng Wu, Qiaoyan Li, Weilun Yin

**Appendix 1. The predictions for small plants derived from MST.**

MST studies subsequent to West *et al.*(1999a, b)^1,2^ have demonstrated that, theoretically, allometric exponents should change gradually with plant size^3,4^, and two major biomass-partitioning patterns have been identified for the largest trees (i.e. MST_t_) and smallest plants at the two ends of this continuum. For small plants, MST predicts that stem mass (*M*_S_) scales isometrically with leaf mass (*M*_L_) and aboveground mass (*M*_A_), i.e. *M*_L_ ∝ *M*_S_ and *M*_S_ ∝ *M*_A_. MST also predicts that height (*H*) scales isometrically with stem diameter (*D*) for small plants, i.e. *H* ∝ *D* ^5,6^. It is known that for any species *M*_S_ =*βρ*_S_*D*^2^*H* (where *ρ*_S_ is stem tissue bulk density)^7^, i.e. *M*_S_ ∝ *D*^2^*H*. For small plants, since *M*_S_ ∝ *M*_A_ and *H* ∝ *D*, then we obtain *M*_A_ ∝ *M*_S_ ∝ *D*^2^*H* ∝ *D*^3^ ∝ *H*^3^. Consequently, we tested the following MST predictions for small plants: *H* ∝ *D*, *M*_L_ ∝ *M*_S_, *M*_A_ ∝ *D*^3^ and *M*_A_ ∝ *H*^3^ (MST_s_ in Table 1).

References

1. West, G. B., Brown, J. H. & Enquist, B. J. The Fourth Dimension of Life: Fractal Geometry and Allometric Scaling of Organisms. *Science*. **284**, 1677-1679 (1999).
2. West, G. B., Brown, J. H. & Enquist, B. J. A General Model for the Structure and Allometry of Plant Vascular Systems. *Nature*. **400**, 664-667 (1999).
3. Enquist, B. J. *et al.* Does the Exception Prove the Rule? *Nature*. **445**, E9-E10, E10-E11 (2007).
4. Niklas, K. J. & Spatz, H. C. Growth and Hydraulic (Not Mechanical) Constraints Govern the Scaling of Tree Height and Mass. *P Natl Acad Sci Usa*. **101**, 15661-15663 (2004).
5. Niklas, K. J. A Phyletic Perspective On the Allometry of Plant Biomass-Partitioning Patterns and Functionally Equivalent Organ-Categories. *New Phytol*. **171**, 27-40 (2006).
6. Niklas, K. J. Modelling Below- and Above-Ground Biomass for Non-Woody and Woody Plants. *Ann Bot-London*. **95**, 315-321 (2004).
7. Enquist, B. J. & Niklas, K. J. Global Allocation Rules for Patterns of Biomass Partitioning in Seed Plants. *Science*. **295**, 1517-1520 (2002).
