# Supplementary material for: Effects of biophysical constraints, climate and phylogeny on forest shrub allometries along an altitudinal gradient in Northeast China
Source: Sci Rep. 2017 Mar 7;7:43769. doi: 10.1038/srep43769 (PMC5339776; doi:10.1038/srep43769)
Supplement: Supplementary Appendix 2 [file srep43769-s2.doc]

**Effects of biophysical constraints, climate and phylogeny on forest shrub allometries along an altitudinal gradient in** **Northeast China**

Authors: Han Sun, Xiangping Wang^*^, Yanwen Fan, Chao Liu, Peng Wu, Qiaoyan Li, Weilun Yin

**Appendix 2.** Summary of mixed-model ANOVA for the effects of climate, site and taxonomic groups on shrub allometric relationships. Here the phylogenetic groups in Table 3? were replaced with taxonomic groups (family, genus and species) to repeat the analysis. Climate includes mean annual temperature (MAT) and precipitation (MAP). Allometric variables were log-transformed prior to analysis.

|  | **df** | **MS** | **P** | **%SS** |  | **df** | **MS** | **P** | **%SS** |
| --- | --- | --- | --- | --- | --- | --- | --- | --- | --- |
| ***H-D*** | |  |  |  | ***M*_L_-*****M*_S_** | |  |  |  |
| log *D* | 1 | 27.96 | 0.000 | 55.81 | log*M*_S_ | 1 | 231.67 | 0.000 | 65.27 |
| MAT | 1 | 1.81 | 0.002 | 3.61 | MAT | 1 | 11.98 | 0.011 | 3.37 |
| MAP | 1 | 0.39 | 0.111 | 0.78 | MAP | 1 | 8.37 | 0.031 | 2.36 |
| Site | 22 | 0.14 | 0.000 | 6.22 | Site | 22 | 1.57 | 0.000 | 9.72 |
| Family | 17 | 0.15 | 0.180 | 4.98 | Family | 17 | 0.90 | 0.091 | 4.33 |
| Genus | 10 | 0.08 | 0.705 | 1.66 | Genus | 10 | 0.39 | 0.039 | 1.11 |
| Species | 18 | 0.12 | 0.000 | 4.20 | Species | 18 | 0.15 | 0.000 | 0.78 |
| log *D*:MAT | 1 | 0.07 | 0.187 | 0.13 | log*M*_S_:MAT | 1 | 0.00 | 0.935 | 0.00 |
| log *D*:MAP | 1 | 0.02 | 0.481 | 0.04 | log*M*_S_:MAP | 1 | 0.01 | 0.840 | 0.00 |
| log *D*:Site | 22 | 0.04 | 0.000 | 1.56 | log*M*_S_:Site | 22 | 0.17 | 0.000 | 1.05 |
| log *D*:Family | 17 | 0.03 | 0.600 | 0.94 | log*M*_S_:Family | 17 | 0.09 | 0.067 | 0.42 |
| log *D*:Genus | 10 | 0.03 | 0.050 | 0.62 | log*M*_S_:Genus | 10 | 0.03 | 0.475 | 0.10 |
| log *D*:Species | 18 | 0.01 | 0.365 | 0.46 | log*M*_S_:Species | 18 | 0.03 | 0.841 | 0.17 |
| Residuals | 799 | 0.01 |  | 19.00 | Residuals | 799 | 0.05 |  | 11.32 |
| ***M*_A_-*H*** | |  |  |  | ***M*_A_-*D*** | |  |  |  |
| log*H* | 1 | 222.93 | 0.000 | 66.33 | log *D* | 1 | 264.42 | 0.000 | 78.67 |
| MAT | 1 | 2.49 | 0.210 | 0.74 | MAT | 1 | 0.25 | 0.521 | 0.07 |
| MAP | 1 | 6.21 | 0.054 | 1.85 | MAP | 1 | 0.11 | 0.674 | 0.03 |
| Site | 22 | 1.50 | 0.000 | 9.79 | Site | 22 | 0.58 | 0.000 | 3.79 |
| Family | 17 | 0.61 | 0.112 | 3.09 | Family | 17 | 0.51 | 0.118 | 2.58 |
| Genus | 10 | 0.29 | 0.067 | 0.85 | Genus | 10 | 0.24 | 0.258 | 0.73 |
| Species | 18 | 0.13 | 0.001 | 0.69 | Species | 18 | 0.17 | 0.000 | 0.94 |
| log*H*:MAT | 1 | 1.30 | 0.036 | 0.39 | log *D*:MAT | 1 | 0.18 | 0.150 | 0.05 |
| log*H*:MAP | 1 | 0.39 | 0.235 | 0.12 | log *D*:MAP | 1 | 0.42 | 0.033 | 0.12 |
| log*H*:Site | 22 | 0.26 | 0.000 | 1.71 | log *D*:Site | 22 | 0.08 | 0.027 | 0.52 |
| log*H*:Family | 17 | 0.14 | 0.314 | 0.72 | log *D*:Family | 17 | 0.07 | 0.949 | 0.36 |
| log*H*:Genus | 10 | 0.10 | 0.405 | 0.31 | log *D*:Genus | 10 | 0.17 | 0.008 | 0.51 |
| log*H*:Species | 18 | 0.09 | 0.032 | 0.50 | log *D*:Species | 18 | 0.05 | 0.491 | 0.25 |
| Residuals | 799 | 0.05 |  | 12.92 | Residuals | 799 | 0.05 |  | 11.37 |
